# Factors, including patient education, that impact cancer treatment adherence in low- and middle-income African countries: a scoping review

**DOI:** 10.1007/s00520-026-11230-8

**Published:** 2026-09-18

**Authors:** Dejen T. Alem, David C. Currow, Patricia M. Davidson, Vanessa N. Brunelli

**Affiliations:** 1 Department of Nursing, College of Medicine and Health Sciences, Debre Markos University, Debre Markos, Ethiopia; 2 Graduate School of Medicine, Faculty of Science, Medicine and Health, University of Wollongong, Wollongong, New South Wales Australia; 3 College of Medicine and Public Health, Flinders University, Bedford Park, South Australia Australia; 4 International Centre for Future Health Systems, Faculty of Medicine and Health, University of New South Wales, Sydney, Australia

**Keywords:** Cancer, Health literacy, Low- and middle-income countries in Africa, Patient education, Treatment adherence

## Abstract

**Purpose:**

Cancer-related morbidity and mortality are disproportionately higher in low- and middle-income countries. One factor that is crucial to improving outcomes is adherence to cancer treatments, which is lower in low- and middle-income countries, especially in Africa, due to patient, provider and system factors. This scoping review aims to map and summarise the characteristics of patient education strategies designed to improve cancer treatment adherence in low- and middle-income African countries.

**Methods:**

This scoping review used the methods of the Joanna Briggs Institute framework. The review included English-language primary studies published in peer-reviewed journals. It focused on adults with cancer receiving an educational intervention aimed at improving treatment adherence in low- and middle-income African countries using narrative synthesis.

**Results:**

Eleven included articles generated three overarching themes on treatment adherence: challenges of social structures; safe, meaningful patient education; and the power of interpersonal interactions between patients, healthcare professionals and families. Technology-assisted education, direct lectures, role playing and demonstration-based methods delivered through group and one-on-one sessions were effective strategies for improving treatment adherence. However, low-income and literacy, treatment complexity and lack of social support were frequently reported as barriers to improving cancer treatment adherence. Challenges of social structure in treatment adherence included low-literacy (and low-health literacy), cultural and religious beliefs, financial burdens, limited access to cancer care and inadequate health system infrastructure.

**Conclusions:**

The mapped evidence highlights that there are several patient education strategies that have been published in low- and middle-income African countries. However, empirical evidence is limited, heterogeneous in findings and not evaluated with validated methods.

## Introduction

Cancer is a global public health issue and the second leading cause of death after cardiovascular disease. In 2022, there were approximately 10 million cancer-related deaths worldwide, with low- and middle-income countries (LMICs) disproportionately higher, accounting for approximately 65% of these deaths [1]. Cancer incidence is also rising globally [2, 3], largely because people are living longer [4] as other acute causes of death are managed more effectively. Ageing remains the greatest single risk factor for cancer [5].

Cancer mortality is also higher in LMICs in Africa, accounting for around 7.8% of global cancer deaths in 2022 [6]. Adherence to cancer treatment refers to the degree to which a patient consistently follows their healthcare provider’s recommendations for cancer care [7]. Adherence rates to cancer treatment in LMICs in Africa are noted to be as low as 20% [8]. Key factors include poorer knowledge of causes of cancer, spiritual beliefs, financial challenges, weaker patient-provider relationships, inability to pay for treatment and fatalistic attitudes [9, 10].

Patient education is a structured, purposeful process through which health professionals provide individuals (and, where appropriate, families or carers) with information, skills and support to enhance their understanding of health conditions, treatments and self-care practices [11]. This strategy is a highly effective approach in influencing treatment adherence in chronic diseases, including cancer [12]. Well-designed education can enhance adherence, ease symptom burden, support lifestyle changes, improve quality of life and ultimately lower premature mortality [9, 10, 13, 14]. Educating patients and caregivers on diagnosis, treatment plans, self-administration of anti-cancer agents, potential side effects, non-pharmacological management, complications and the importance of attending clinic for all appointments has the potential to improve patient outcomes [15].

Beyond the African context targeted by this review, evidence from other global LMICs highlights that patient education has been shown to effectively improve health outcomes for oncology patients, including enhanced quality of life and reduced premature mortality [13, 16]. A 2023 review by Christiansen et al. [13] found that technology-based interventions (especially interactive education videos) were the most frequently utilised and effective strategies, particularly for populations with low literacy, by enhancing cancer knowledge [13]. A 2022 systematic review by Chauke et al. reported that patient knowledge of disease and medication are one central mediating factor [10], while Kostantinou et al. showed that digital education combining disease and medication information, motivational interviewing, reinforcement and motivational messages leads to improvements in treatment adherence [17]. Such work requires consideration and understanding of literacy and health literacy in LMICs.

Most evidence in the context of LMICs has reported either on educational strategies [18] or factors that influence cancer treatment adherence [19], but not on the discrete context of the role of patient education on cancer treatment adherence. This is a gap in our knowledge of what constitutes successful patient education relative to improved cancer treatment adherence. Hence, this review builds on the recent reviews of Christiansen et al. [13] and Chauke et al. [10]. This scoping review aimed to map the literature on patient education specific to LMICs in Africa, with a particular focus on studies that identify cancer treatment adherence as a primary outcome.

### Review questions

1. What are the characteristics of the content and mode of delivery of patient education that aim to improve cancer treatment adherence in LMICs in African countries?
2. What factors influence the success and implementation of patient education to improve cancer treatment adherence in LMICs in African countries?

## Methods

### The study protocols

This scoping review was guided by the Joanna Briggs Institute (JBI) methodology for scoping reviews [20] and the findings were reported in accordance with the Preferred Reporting Items for Systematic Reviews and Meta-Analysis extension for Scoping Reviews (PRISMA-ScR) [21]. The protocol for this study is registered in the PROSPERO database under the registration ID 1021376.

### Inclusion criteria

The focus of this scoping review was refined with the ‘population-concept-context’ mnemonic of the JBI methodology [22]:

### Population

This scoping review considered studies conducted among adult (18 years or older) patients in LMICs in Africa (based on World Bank income classification, LMICs include low, lower middle and upper middle income), diagnosed with cancer (any type, any stage in the cancer continuum) and prescribed anti-cancer treatment (any kind) and peer-reviewed journals published in English.

### Concepts

The concepts of interest for this review were related to:

I. Patient education, including type of content (e.g. clinical, informational, psychological/emotional, social, spiritual), intervention(s) or strategies (e.g. tailored, general), delivery modes (e.g. technology-assisted/Short Message Service, video), and education method (e.g. 1:1 motivational interviewing, group education).
II. Cancer treatment adherence including elements of social determinants of health (e.g. income and social protection, education, job (in)security and unemployment, working life conditions, food insecurity, housing, social inclusion, structural and social conflict, access to quality care) and other determinants (e.g. sex, religion, culture, health literacy, health system, healthcare provider).

### Context

This review considered studies that reported on the implementation and evaluation of patient education, which had a primary outcome of, or focus on, cancer treatment adherence in inpatient or outpatient settings in LMICs in Africa.

### Sources

This scoping review considered peer-reviewed publications in English. Studies using quantitative, qualitative and mixed-methods research designs were considered for inclusion. Eligible quantitative studies were experimental, observational or descriptive cross-sectional designs. Qualitative studies included in the review were interview or focus group designs, or qualitative descriptions of interventions. No restrictions were placed on the duration of follow-up. Studies were excluded if they did not focus on the two key concepts of interest: patient education or educational interventions, and cancer treatment adherence, including factors influential of adherence, as well as the grey literature.

### Search strategy

An initial limited search of MEDLINE (PubMed) was undertaken to identify articles on the topic. The text words and key terms contained in the titles and abstracts of relevant articles, and the index terms used to describe the articles, were used to develop a full search strategy to be employed across all included databases. To enhance specificity and sensitivity, the ‘explode’ command, the truncation symbol (*), and Boolean terms (AND, OR) were used to combine the different key search concepts (patient self-management education; cancer treatment adherence; low- and middle-income Africa) (Table 1).

**Table 1:** Presents the final keyword search as employed for PubMed, with initial search results

Search strategy	Number of articles generated
(((((((((((((((((((((Self-management education[MeSH Terms]) OR chemotherapy adherence[MeSH Terms]) OR (low[MeSH Terms] AND middle income countr[MeSH Terms])) AND self-management[Text Word]) OR self-management strateg*[Text Word]) OR education[Text Word]) OR self-care[Text Word]) OR self-care[Text Word]) OR self-care management[Text Word]) OR cancer care[Text Word]) OR supported care[Text Word]) OR chemotherapy[Text Word]) OR adherence[Text Word]) OR treatment adherence[Text Word]) OR treatment[Text Word]) OR adherence[Text Word]) OR patient education[Text Word]) OR cancer education[Text Word]) OR cancer counseling[Text Word]) OR cancer[Text Word]) OR cancer counseling[Text Word]) AND developing countr*[Text Word]	**568**

Four electronic databases were searched from their inception to March 2025, including the Cochrane Library, MEDLINE through PubMed, CINAHL and Web of Science. To identify unindexed articles in the searched databases, literature was searched using Google Scholar, as well as manual searches of reference lists of relevant articles in the field. Websites were also searched, including the World Health Organisation, Union for International Cancer Control, International Cancer Foundation, International Agency for Research on Cancer, American Cancer Society and the Agency for Healthcare Research and Quality.

### Study selection

All records were exported into EndNote software version 20.0 [23]. Duplicates and documents assessed irrelevant with a ‘first pass’ rapid review by two authors (DTA and VNB) were removed.

Guided by the inclusion criteria, two authors (DTA and VNB) independently screened the full-text versions to determine the final number of included articles. Where there was reviewer disagreement between DTA and VNB, these authors met to discuss the study in question and together re-read the full-text version. The advice of the wider research team (DCC and PMD) helped to resolve any disagreements.

### Methodological quality assessment

The methodological quality of the included studies was assessed using the Mixed Methods Appraisal Tool (MMAT). The MMAT was selected because it provides criteria appraising studies with different methodological designs, including quantitative, qualitative and mixed-method studies. The two screening questions and five design-specific appraisal criteria were applied according to the methodological design of each included study [24].

### Data extraction

Informed by the JBI data extraction template [22] and our review questions, a data abstraction form, in Microsoft Excel format, was developed. Other information collected included geographic locations of studies, study settings and designs, research participants’ involvement and the interventions and strategies tested relative to cancer treatment adherence and education delivery modes. Any disagreements about data extracted were discussed between the two authors (DTA and VNB) and, when necessary, with the wider research team (DCC and PMD) until consensus was reached. Table 2 presents the data extraction form. Given the complexity of implementation studies and the heterogeneity in implementation components and study designs, appraisal of the quality of studies was not undertaken. This is consistent with other similarly designed systematic scoping reviews [25].

**Table 2:** Data extraction form

Studies included	Year	Country of origin	Country income level	Study design	Research aim/s	Primary outcome	Data collection methods	Validated tool/s used for primary outcome measurement	Target education participant	Study population (age)	Study population (cancer type, disease stage, phase in cancer continuum)	Management education intensity (implementation period/duration, frequency/follow-up)	Education provider (nurse, allied health, researcher, other)	Key findings that relate to the research aim/questions	Participants	Prevalence/effect size
[37]	2022	Mali	Low	Survey	Low health literacy is a leading cause of treatment abandonment among patients	Patients knowledge	Questionnaire	Self-administered questionnaire developed by researchers (not validated)	Cancer patients receiving care	Adults	All type	85 min	Nurses	Sub-Saharan Africa needs standardised patient education	100	*p* = 86%
[30]	2022	Ethiopia	Low	Hospital-based prospective cross-sectional study design	Aimed to assess chemotherapy adherence and associated factors among patients with cancer	Adherence to chemotherapy	Review of medical records and a face-to-face interview	MMAS-8	Cancer patients	All age groups	Any type			Chemotherapy treatment adherence was 42.3% & should be improved through health education	460	*p* = 42.3%
[34]	2020	Kenya	Lower middle	Cluster randomised controlled trial, qualitative design	Improving understanding of cervical cancer, screening importance and self-collection steps	Women’s experiences with cervical cancer outreach and education strategy	In-depth interview	Interviews developed by the researchers (not validated)	Women	Adults only (women aged 25–65 years)	Cervical Cancer	15 min for group intervention, 10 min for one-on-one intervention	Trained CHVs, research team, nurses	Health workers can help bridge the gap between cervical cancer screening uptake and acceptability	525	N/A
[32]	2021	Tanzania	Lower middle	Cross-sectional educational intervention	The impact of education on baseline human papillomavirus and cervical cancer knowledge	Cervical cancer knowledge	Survey questionnaire	Survey developed by researchers (not validated)	Women	Women aged 18 +	Cervical cancer	15-min video watching	Researcher	A short video-based educational intervention improved baseline knowledge	825	Mean increase = 1.64
y[29]	2012	Ethiopia	Low	Institutionally based cross-sectional descriptive study design	To assess treatment compliance and associated factors among cervical cancer patients	Treatment compliance	Face-to-face structured questionnaire-based interview	Questionnaire developed by the researchers (not validated)	Women	Adults	Cervical cancer			30.3% of cervical cancer patients were non-compliant with the treatment services	314	*p* = 69.7%
[31]	2018	Ethiopia	Low	Institution-based cross-sectional study design	To assess self-care behaviour among cancer patients who are on chemotherapy treatment	Self-care behaviour	Structured questionnaire with face-to-face interview	The Self-Care Behaviour Questionnaire and the Self-Care Utilization	Cancer patients	Adults (> 18 years) who are on chemotherapy only	All type			Self-care behaviour was insufficient, and intensive awareness Creation campaigns are needed	231	*p* = 42%
[38]	2019	Rwandan	Lower middle	A pre- and post-test design	To increase patient knowledge by implementing a standardised oncology education programme	Patients knowledge	Pre- and post-test design developed by the study team	Cancer and You education booklet created by Global Oncology	Cancer patients	Adults only (43 to 60 years)	All type	5 months	Nurses	standardised education significantly improved patient knowledge	40	*p* = 19%
[33]	2019	Tanzania	Lower middle	Surveys	Educating Maasai women on cervical cancer and screening	Women’s knowledge	Pre- and post-intervention surveys		Women, men, healthcare workers	Women (30–49 years old)	Cervical cancer	Six weeks	Researcher	Women showed an increase in knowledge following education	66	Mean increase = 21.4%
[35]	2022	Kenya	Lower middle	Qualitative study design	To identify and understand barriers and facilitators to chemotherapy initiation and adherence	Barriers and facilitators to chemotherapy initiation and adherence	Interviews	Situated Information Motivation behaviour model	Patients with HIV-associated Kaposi’s sarcoma	Adults only (> 18 years)	Kaposi’s sarcoma			Findings support efforts to enhance cancer education and access	57	N/A
[39]	2009	South Africa, Uganda	Upper middle, low	Semi-structured qualitative interview study	To explore the information needs of patients with incurable disease & their family caregivers	Information about the needs of cancer patients	Face-to-face interviews		Patients, caregivers		Incurable progressive disease (cancer, HIV, motor neuron disease)			Cancer patients & carers lacked information regarding incurable disease	90	N/A
[36]	2024	Kenya	Lower middle	Multisite cluster randomized trial	To evaluate the effectiveness of short message service support on adherence to chemotherapy treatment	Adherence to chemotherapy treatment	Questionnaire	Medication possession ratio tool	General cancer survivorship: self-care and reminders about keeping clinic appointments	Adults only	All types		Clinical psychologists	Adherence was 83%. Women were perfect adherents than males. Perfect adherers were satisfied with SMS support	538	ES (Cohen’s *d*) = 0.6

*P* prevalence, *ES* effect size, *N/A* not applicable

### Data analysis and narrative synthesis approach

Whilst there is a lack of consensus regarding the best approach for synthesising scoping review findings, narrative synthesis remains a recommended approach [25] including within the JBI literature review methodology [26]. This is an approach to the synthesis of evidence relevant to a wide range of issues [27] by using words and text to ‘tell the story’, that is, to summarise and explain the results of several investigations [28]. In this review, tabulation, textual descriptions and thematic analysis utilising concept mapping, as suggested by Popay et al*.* [27], were employed iteratively by the research team.

## Results

### Search results

**Fig. 1:**
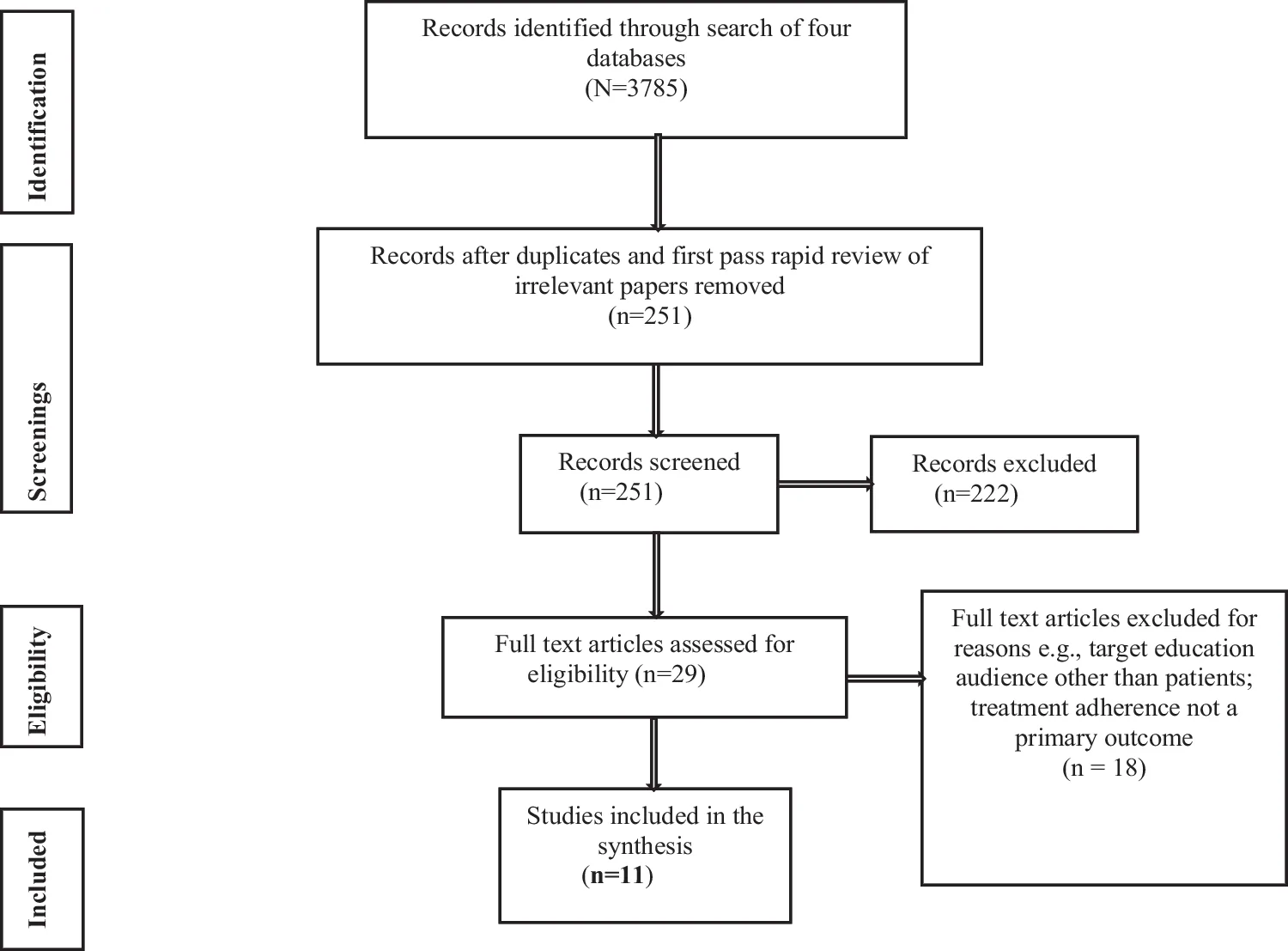
PRISMA flowchart

A total of 3785 studies were obtained through database retrieval and reference tracing. After removal of duplicates and documents assessed as irrelevant with a ‘first pass’ rapid review, 251 articles underwent title and abstract screening. This process further excluded 222 articles, reducing the number of eligible articles for full-text review to 29. Of the 29 full-text articles assessed for eligibility, 11 articles were included to comply with the inclusion criteria (Fig. 1).

### Characteristics of included studies

Among the 11 included studies (Table 3): three were conducted in Ethiopia [29–31]; two in Tanzania [32, 33]; three in Kenya [34–36] and one each in Malawi [37], Rwanda [38], South Africa and Uganda [39]. The majority of the studies was published after 2018 [30–35, 37, 38], with two studies published between 2012 and 2016 [29, 40], and one study published before 2010 [39].

**Table 3:** Overview of included studies

Author Year	Country	Design	Target audience	Intervention	Sample size	Education provider	Main findings
[37]	Mali	Survey	Adult cancer patients	Video	100	Nurses	Standardised patient education is important for sub-Saharan Africa
[30]	Ethiopia	Cross-section	Adult cancer patients	N/A	460	Not mentioned	Chemotherapy treatment adherence was 42.3%. Suggested improvements with culturally sensitive health education
[34]	Kenya	RCT, qualitative	Women with cervical cancer	Scripts, visual prompts, messaging, flipcharts, posters, brochures	525	Nurses	Health workers helped bridge the gap between cervical cancer screening uptake and treatment acceptability
[32]	Tanzania	Cross-section	Women with cervical cancer	Video	825	Researcher (public health educators, physicians)	A short video-based educational intervention improved baseline knowledge
[29]	Ethiopia	Cross-section	Women with cervical cancer	N/A	314	Not mentioned	30.3% of cervical cancer patients were non-compliant with treatment. Poor understanding of treatment advantages was one of the factors
[31]	Ethiopia	Cross section	Adult cancer patients	N/A	231	Not mentioned	Self-care behaviour was lacking. Suggested intensive awareness campaigns are needed
[38]	Rwanda	Pre-post	Adult cancer patients	Lecture, demonstration and role playing	40	Nurses	Standardised education significantly improved patient knowledge and understanding
[33]	Tanzania	Survey	Women with cervical cancer	Lecture, poster	66	Researcher (Physicians)	Participants' knowledge increased post-education intervention
[35]	Kenya	Interview	Kaposi’s sarcoma patients	N/A	57	Not mentioned	Efforts to enhance cancer education improve patient awareness and compliance
[39]	South Africa and Uganda	Interview	Adult cancer patients	N/A	90	Not mentioned	Cancer patients and carers lacked information regarding the incurability of the disease. Suggested disease- and treatment-specific education and information are critical
[36]	Kenya	RCT	Adult cancer patients	SMS	538	Clinical psychologist	Adherence was 83%. Women were more compliant than men. The SMS reinforcement intervention was well received

N/A = observational studies with no intervention

The 11 studies included a total of 3246 participants, the majority of whom had cervical cancer, followed by breast cancer (this was because some of the included studies focused exclusively on breast and cervical cancer). The participant sample sizes ranged from 40 to 825 participants. The mean ages ranged from 18 to 66 years. All 11 studies included people with cancer across all stages, cancer types and treatment phases.

**Fig. 2:**
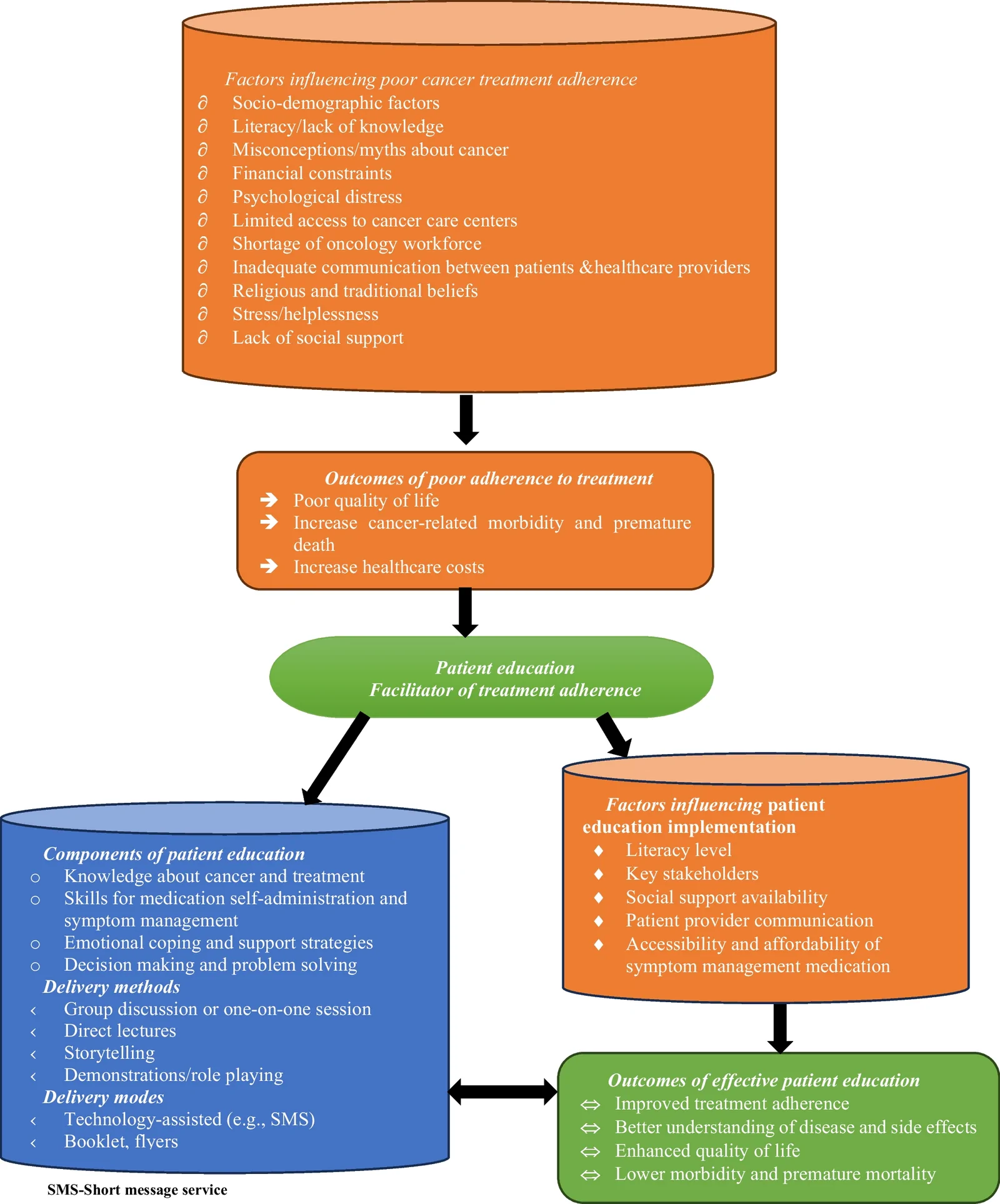
A concept map generated from review findings showing the factors influencing cancer treatment adherence and the role of patient education in improving treatment adherence

There were six experimental studies [32–34, 36–38], two qualitative studies [35, 39] and three observational studies [29–31]. Most of the studies employed a quantitative design, while two [35, 39] were purely qualitative, and one experimental study [34] adopted a mixed-methods design that included a qualitative component. Of the experimental studies, two were randomised controlled trials (RCTs) [34, 36], while the remaining four used a pre- and post-test design [32, 33, 37, 38]. All experimental studies, including the mixed methods design which incorporated a qualitative design, were evaluation studies aimed at assessing the effectiveness of interventions through pre- and post-intervention assessments. The following text explicates Fig. 2, a concept map of the intersections between factors of cancer treatment adherence and the influences of patient education on improving adherence [30, 32, 35, 36, 39].

### Methodological quality assessment

All included studies met the two MMAT screening criteria, with none receiving a response of ‘no’ or ‘can’t tell’. This indicates that all studies had clearly defined research questions and that collected data were considered appropriate for addressing their research objectives. The other five criteria were based on the study design and were used to assess the methodological quality of the included studies. Qualitative studies generally meet all MMAT criteria. Quantitative descriptive studies (observational studies) also demonstrated good methodological quality; however, two studies [29, 31] were assessed as ‘can’t tell’ regarding whether their samples were representative of the target population, as both studies were conducted in a single hospital. Quantitative non-randomized intervention studies had some methodological limitations, including unclear reporting of participant representativeness [33, 37, 38, 41], lack of adjustment for potential confounding factors [33, 37, 38] and unclear reporting of completeness of outcome data [33, 37] (Table 4).

**Table 4:** Methodological quality appraisal of included studies using the mixed methods appraisal tool

Study	Study design	S1	S2	Q1	Q2	Q3	Q4	Q5
Tilly, Alyssa E., et al. [37]	Quantitative non-randomised intervention design	Y	Y	CT	Y	CT	N	Y
Bekalu YE, Wudu MA, Gashu AW [30]	Quantitative descriptive (observational)	Y	Y	Y	Y	Y	Y	Y
Choi, Yujung, et al. [34]	Mixed (non-randomised intervention design and qualitative)	Y	Y	Y	Y	Y	Y	Y
Cooper, Emma C., et al. [32]	Quantitative non-randomised intervention design	Y	Y	Y	Y	Y	Y	Y
Gebre, Y., et al. [29]	Quantitative descriptive (observational)	Y	Y	Y	CT	Y	Y	Y
GEBRE, TSIGE. [31]	Quantitative descriptive (observational)	Y	Y	Y	CT	Y	Y	Y
Habimana, Olivier, et al. [38]	Quantitative non-randomised intervention design	Y	Y	CT	Y	Y	N	Y
Lidofsky, A., et al. [33]	Quantitative non-randomised intervention design	Y	Y	CT	Y	CT	N	Y
McMahon, Devon E., et al. [35]	Qualitative design	Y	Y	Y	Y	Y	Y	Y
Selman, Lucy, et al. [39]	Qualitative design	Y	Y	Y	Y	Y	Y	Y
Mchidi NK, Oyore JP, Ogweno G. [41]	Quantitative non-randomised intervention design	Y	Y	CT	Y	Y	N	Y

*S* screening question, *Q* quality approval criteria (design specific), *Y* yes, *N* no, *CT* can’t tell

### Responding to the review questions

Education methods included:

- direct lecture, role playing and demonstration (*n* = 2 studies) [33, 38];
- group education sessions facilitated by a researcher or healthcare professional (*n* = 4 studies) [32, 33, 37, 38];
- 1:1 education sessions using motivational interviewing (*n* = 2 studies) [36,; or
- a combination of group education, either preceded or followed by a 1:1 session (*n* = 1 study) [34].

The intensity or duration of education ranged from 2 days to 5 months, with only one study reporting an intervention of five months’ duration [38]. Most studies ranged from minutes to 3 weeks of intervention duration [32–34, 37].

The delivery modes of patient education reported in the studies included in this review focused largely on technology-assisted interventions or strategies, such as:

- internet-based support interventions (*n* = 2 studies) [34,;
- videos (*n* = 2 studies) [32, and
- a full-process information management system (*n* = 1 study) [34].

### Characteristics of patient education to improve treatment adherence in LMICs in Africa

The content of patient education interventions and strategies reported in the literature, contextual to LMICs in Africa, is varied, including information support about tumour-specific disease and treatment knowledge, available aid resources or organisational service information, outpatient consultation arrangements and public lectures [33, 34, 36–39].

Nurses were the most frequent patient education provider (*n* = 3 studies) [34, 37, 38], followed by allied healthcare professionals (*n* = 2 studies) [34, 36]. Three studies reported the researcher (master’s degree holders) as the role to facilitate patient education [32–34].

Qualitative studies utilised one-on-one semi-structured interviews of varying duration [34, 35, 39] to assess barriers and facilitators [35, 39] and also to evaluate programme effectiveness [34]. Observational studies employed cross-sectional designs to explore factors and behaviours influencing cancer treatment adherence [30, 31].

### Factors influencing the implementation and uptake of patient education in LMICs in Africa

Multiple and often competing factors influenced the success of patient education for adults undergoing cancer treatment in LMICs in Africa [42]. Individuals with higher levels of income, for instance, reported better access to different anti-cancer and supportive care medications and resources necessary for effective cancer management [43]. Similarly, patients with higher education levels appear to have better understandings of their cancer condition and treatment options, are more informed in their decision-making and report more effective day-to-day management [44].

### Narrative synthesised findings

Relative to each review question, key concepts supported by textual description were constructed through the qualitative analytical approach, with an overarching theme derived. Table 5 presents the analytical findings.

**Table 5:** Narrative synthesised findings of cancer treatment adherence and patient education in low and middle-income African countries

Key concepts, supported with textual description	Themes
*Appropriate education:* Culturally sensitive materials, standardised yet tailored, visual (video, picture, written…), and focused on both disease- and treatment-specific content, delivered with purposeful engagement by health professionals, is important. Appropriate education delivered by nurses offers the potential to enhance clinical and quality outcomes	Meaningful education to improve patient cancer treatment, understanding and adherence
*Engagement and interactions:* Enhancing patient, caregiver and health professional awareness regarding supported self-management in cancer care is a key first step to improving cancer treatment adherence and outcomes. Purposeful engagement by health professionals, caregivers and families must underpin interactions with the person with cancer to positively influence treatment adherence	The power of interpersonal interactions between nurse, patient and family on cancer treatment adherence
*Tensions and contradictions:* Social, cultural and structural barriers, including financial implications, healthcare access and infrastructure challenges, cultural and religious beliefs and education and health literacy can significantly impact the ability, capacity and intention of the person with cancer to start—and continue—treatment	The challenge of social structures on cancer treatment initiation and adherence

## Discussion

This scoping review aimed to map the available evidence on factors including patient education that impact cancer treatment adherence in LMICs in Africa. The review identified three overarching themes: (1) safe and meaningful patient education, (2) challenges of social structures and (3) the power of interpersonal interactions among people living with cancer, healthcare professionals and family caregivers. These findings reveal that improving treatment adherence requires more than changing individual patient behaviour.

### Patient education to improve cancer patient treatment, understanding and adherence

In LMICs, several studies that have explored cancer treatment adherence highlight health education as a key approach to improving adherence [13, 30]. For instance, a study conducted in Ethiopia [30] suggested the importance of improving patients’ understanding of their illness and treatment through health education programmes; however, the authors did not specify content or what type of educational approach would be most suitable in this context. Yates et al*.* [12] and Brunelli [45] emphasise that patient education, an active and participatory process, plays a crucial role in improving health outcomes among people with cancer and chronic diseases, as long as the education delivered is personalised and tailored to the context and situatedness of the patient and their support people. Some studies from LMICs support this view, underscoring the need for standardised yet tailored patient education, though they stop short of making explicit what might comprise appropriately tailored education [37].

Research by Lidofsky et al*.* and Howell et al*.* [33, 46] align with the positions of Yates et al. [12] and Bruneli [45], concluding that tailored patient education programmes are associated with improved adherence, which, in turn, enhances cancer outcomes and other health-related outcomes, such as quality of life [33, 46]. Moreover, such programmes not only empower people affected by cancer by increasing their knowledge about the disease and its treatment, but also enhance the capacity of health care professionals in delivering more effective person-centred care [33, 47].

A variety of patient education intervention delivery modes have been utilised in LMICs. A 2024 study in Kenya that aimed to improve patients’ understanding of treatment recommendations evaluated the effectiveness of short message service support and found that this delivery mode intervention significantly enhanced treatment adherence [36].

Christiansen et al. [13], in their systematic review, on the other hand, revealed that technology-based interventions, particularly interactive video-based learning, were among the most common and effective education methods. They stated that visual components are especially valuable in populations with limited literacy, which is a prevalent issue in LMICs. Such approaches not only improve patients’ knowledge but also help them engage with information that aligns with their needs and preferences [13]. This is important in rural areas where lower education levels and poor literacy and health literacy often contribute to misconceptions about cancer, poor perceptions of treatment and difficulties in understanding complex medical conditions [37, 48].

Kostantinou et al*.* [17] reported that motivational interviewing, regular reinforcement and motivational messages, as methods to deliver patient education, lead to improvements in treatment adherence. Similarly, Chauke et al*.* [10] supported these strategies but noted that their success largely depends on addressing literacy barriers and communication challenges within LMICs, as well as on developing policies that facilitate effective patient-provider communication. To address these challenges, educational materials should be both culturally sensitive and standardised, while allowing for adaptation to the local contexts.

### The power of interpersonal interactions between patients, healthcare professionals and family on cancer treatment adherence

Relationships among healthcare professionals, people living with cancer and family caregivers play a critical role in effective cancer care. Moreover, active engagement and communication between these groups is integral in facilitating the abilities of individuals with cancer to manage, thereby improving the likelihood of treatment adherence. Several studies [35, 36, 39, 49] highlight that strong interpersonal communication and collaboration among these stakeholder groups help patients better understand their illness, recognise the importance of adhering to treatment and anticipate potential side effects. Research by Selman et al*.* [39] and Habimana et al*.* [38] demonstrated that effective communication between patients, family caregivers and healthcare providers improves trust, shared understanding and emotional support, all of which serve to positively influence cancer treatment adherence. This is consistent with evidence from high-income countries, where quality interactions have been shown to improve cancer care outcomes [50–52].

However, in LMICs in Africa, several challenges hinder the development of such meaningful interactions. These include limited knowledge of cancer care among healthcare professionals, including negative perceptions about treatment options, efficacy and outcomes and socio-cultural factors that constrain the delivery of effective patient and family education [39, 52, 53]. McMahon et al*.* [35] also noted that many individuals living with cancer in LMICs in Africa often perceive cancer as an incurable condition, which undermines motivation for treatment commencement, let alone adherence. Similarly, family caregivers can have insufficient understanding of cancer treatments, hold unfavourable attitudes towards medical care and, sometimes, adhere to religious or cultural beliefs that weaken confidence in medical interventions [30, 39, 54].

To address these challenges, evidence from LMICs in Africa consistently emphasises the importance of culturally sensitive and contextually appropriate patient education programmes [33, 38, 55, 56]. Such programmes can strengthen the cooperation between patients, family caregivers and healthcare professionals by improving communication, trust and shared responsibility in care. Further exploration of cancer treatment experience that involves diverse stakeholders is vital.

### The challenge of social structures on cancer treatment initiation and adherence in LMICs in Africa

Factors influencing cancer treatment initiation and adherence in LMICs in Africa are multifaceted and often intersect, including literacy, cultural and religious beliefs, economic burden, healthcare access (such as distance to health facilities) and health system infrastructure challenges [29, 30, 35, 39]. These are explained in greater depth below:

#### Literacy

Literacy is the ability to identify, understand, interpret, create and communicate using written materials [57]. Health literacy, which is a component of literacy, is the ability of individuals to understand and use health-related information in ways that promote and maintain good health [58]. Digital literacy also refers to the ability to use technologies to access, evaluate and communicate information [59]. In many African countries, low health literacy hinders patients’ understanding of cancer, its treatment and the importance of adhering to treatment, fostering myths that cancer is incurable [35] and undermining informed decision-making [29]. These misconceptions contribute to reduced adherence, with some patients discontinuing treatment after it has begun [56, 60, 61].

#### Cultural and religious beliefs

In LMICs in Africa, cultural and religious beliefs strongly shape how cancer patients view their illness, treatment and healthcare choices. Traditional medicine and spiritual practices often take precedence over modern evidence-based medical care, leading some patients to delay, discontinue or forgo cancer treatment in favour of alternative therapies such as holy water or traditional healers [29, 30, 35], which can worsen outcomes [56, 62–64].

#### Economic burden

In LMICs in Africa, the financial burden of cancer severely limits access to and continuity of treatment [35]. High cost for care, medications, transport and lost wages, often compounded by lack of health insurance, create major barriers [29], leading many to seek financial help through social media, while others face these challenges without external support [65–68].

#### Healthcare access and health system infrastructure challenges

In LMICs in Africa, gaps in healthcare access and health system infrastructure hinder cancer treatment initiation and adherence. The limited or absence of facilities, shortages of trained oncology staff, poor transport in rural areas, overcrowding, long waiting times and limited specialised oncology services in urban settings all create significant barriers to care [35, 56, 65].

Collectively, these findings demonstrate that treatment initiation and adherence are shaped by interconnected social and structural factors rather than by individual patient behaviour alone [69]. While the existing literature widely acknowledges that adherence is governed by this complex interaction of socioeconomic, cultural and structural factors [29, 30, 35, 70], these studies suffer from both notable heterogeneity and methodological limitations. Primarily, prior research has relied on divergent measurement tools and evaluated outcomes almost from the patient’s perspective only. In addition, the methodological quality appraisal indicated that although most included studies were of generally good methodological quality, some non-randomised studies had limitations related to participant representativeness, adjustment for confounding factors and completeness of outcome data.

Studies in Ethiopia (2015; 2023), for example, employed a cross-sectional design and self-administered questionnaires targeting only people living with cancer in a hospital setting [29, 30]. The key factors identified in this study were related primarily to sociocultural related to sociocultural influence. A study conducted in Kenya [35] employed a qualitative study design, focused solely on patients and identified several barriers to adherence, including lack of financial means, difficulty with the convenience of appointments, such as distance to facility, appointment times, long lines, limited appointments and lack of proper or sufficient information about chemotherapy. Notably, this study excluded other important stakeholders, such as family caregivers and health care professionals, whose perspectives might have contributed to a more comprehensive understanding of the issue.

These findings are consistent with the literature from LMICs beyond Africa [10]. Studies conducted in other LMICs have similarly reported that poor treatment adherence is influenced by a combination of lack of knowledge about cancer and its treatment, negative attitude towards cancer care, limited access and cultural and religious beliefs. These findings suggest that the factors to treatment adherence identified in this review are not unique to African settings but are common across many resource-constrained settings. In sharp contrast, studies from high-income countries report that although socioeconomic inequalities remain important, patients benefit from stronger health systems, more comprehensive insurance coverage and greater availability of patient education and supportive care services which may help improve treatment adherence [71].

### Limitations of the study

This scoping review is subject to several limitations including (1) limited number of available articles addressing patient education and cancer treatment adherence in LMCs in Africa, limiting the comprehensiveness of findings, (2) methodological differences in the included studies, such as different study objectives and outcome measures which complicate the ability to draw uniform conclusions, (3) the inclusion of English-only studies and exclusion of grey literature may have resulted in the omission of relevant articles and (4) most studies lacked long-term data and standardised tools to measure treatment adherence, limiting insights into the effectiveness of patient education. Addressing these gaps in future research will be important to developing a more comprehensive understanding of the role of patient education in improving cancer treatment adherence in African LMICs. In addition, this review does not include individuals who do not access cancer care, a population about which little is currently known.

### Implications for research

In LMICs in Africa, patient education arguably represents a critical role in improving cancer treatment adherence and outcomes for people with cancer. However, the most effective educational approach to improve cancer treatment adherence among the African population is unclear. Future research should consider context-specific, evidence-based, tailored educational interventions developed in collaboration with African people with cancer. Educational interventions should be co-designed, including diverse stakeholder groups, specifically people with cancer, family caregivers and healthcare professionals. Studies must aim to confirm or refute previously identified factors to develop a comprehensive understanding of cancer treatment adherence. To this end, longitudinal studies will be necessary to determine the efficacy of patient education programmes on cancer treatment adherence and longer-term health-related outcomes, for instance, quality of life.

## Conclusion

The mapped evidence highlights that patient education is frequently used and associated with the potential for improving cancer treatment adherence in the African region. However, treatment adherence is influenced by multiple interconnected factors, such as limited health literacy and cultural and religious beliefs. The review also highlighted that the success of patient education intervention is also shaped by factors such as effective patient-provider communication and literacy level. Despite the potential, empirical evidence is limited, and the available evidence is heterogeneous in its objectives, design and outcomes. This methodological heterogeneity hinders a comprehensive understanding of the content, modes and methods of education delivery required to affect cancer treatment adherence, underscoring the need for further research.

## Data Availability

No datasets were generated or analysed during the current study.
